# Demographic and Lifestyle Factors Associated with Patient-Reported Acute COVID-19 Vaccine Reactivity

**DOI:** 10.3390/vaccines11061072

**Published:** 2023-06-07

**Authors:** Andrew George, Haley M. Goble, Smaran Garlapati, Shari R. Liberman, Bradley S. Lambert

**Affiliations:** Houston Methodist Orthopedics and Sports Medicine, Houston, TX 77030, USA

**Keywords:** COVID-19 vaccine, vaccine hesitancy, vaccine reactivity

## Abstract

Patient-reported vaccine reactivity (PRVR) is a major contributor to COVID-19 vaccine hesitancy. PRVR responses to the COVID-19 vaccine may be affected by several modifiable and non-modifiable factors that influence immune function. Understanding the effects of these factors on PRVR can aid in better educating patients on expectations, as well as formulating public health strategies to increase the levels of community vaccination.

## 1. Introduction

The COVID-19 pandemic has caused over 6 million deaths worldwide [1], leading to the development of several vaccines in an unprecedented, short period of time. Over 5.5 billion people have received a COVID-19 vaccine [2], which translates to over seventy percent of the global population. Vaccination has been shown to be highly effective in preventing and mitigating the deleterious effects of COVID-19 infection [3], but over one third of the global population is not fully vaccinated [2]. While the reasons for vaccine hesitancy are multifactorial, patient-reported vaccine reactivity (PRVR) was a major factor in a multinational study evaluating the reasons for vaccine hesitancy in 44,000 individuals; a concern about adverse effects was the most common reason for vaccine hesitancy across all demographics [4]. Common adverse effects of the vaccine included fatigue, muscle pain, joint pain, headache, and fever. Furthermore, health care workers were noted to be the most trusted source of guidance about COVID-19 vaccines. It is therefore imperative for health care providers and public health workers to understand which patients are more likely to develop adverse effects in order to better counsel patients regarding the vaccine, and decrease vaccine hesitancy in the community.

The majority of research on COVID-19 vaccine adverse effects has been conducted through randomized clinical trials prior to vaccine roll-out, but there have been fewer studies evaluating real-world patient-reported data to determine which patients may be at increased risk of experiencing adverse effects. Therefore, we sought to identify factors associated with adverse effects after COVID-19 vaccination. We hypothesized that factors associated with a less robust immune system would be associated with decreased PRVR.

## 2. Materials and Methods

The following procedures were approved by our Institutional Review Board for research involving human participants (Protocol 00030244). Following consent, an electronic survey (RedCap, Version 10.5.2) was completed by 12,890 adults [64 ± 15 yrs. (18–98), ♂5672♀7132] who had received their 2nd vaccine dose (Pfizer–BioNTech) between January 2021 and December 2021 through a single hospital system in the Texas Medical Center (Houston, TX, USA). Patients were asked to report the incidence and severity ratings (SR, electronic visual analogue scale, none–severe, 0–10) of PRVR (injection site pain, nausea/vomiting, fever, body ache, joint pain, fatigue, headache, others) for each dose [5]. Demographic information, including age, height, weight, sex, and ethnicity, was obtained. Participants provided their medical history including blood type, history of diabetes, cardiopulmonary conditions, heart attack, stroke, hypertension, hypothyroidism, hyperlipidemia, rheumatoid arthritis, asthma, cancer, and any autoimmune conditions. Lifestyle habits were assessed, including smoking, alcohol consumption, and physical activity. Information gathered on physical activity included frequency of planned exercise, weight training, and aerobic exercise (days per week). Chi-square analysis was used to compare the incidence of PRVR among survey subgroups. Multiple logistic regression was used to determine symptom frequency odds ratios among survey subgroups. Correlation analysis was used to characterize the relationship between demographics, modifiable lifestyle factors, and PRVR severity with a minimum r value requirement of 0.1 as r values were interpreted as follows: <0.1 (negligible), 0.1–0.3 (weak), 0.3–0.7 (moderate), and >0.7 (strong) [6]. Type I error was set at α = 0.05 for all analyses. 

## 3. Results

Sample population demographics, comorbidity frequencies, and modifiable lifestyle factors are presented in Table 1. 

The incidence of PRVR was higher following the second dose (^d1^33.6%, ^d2^42.2%; *p* < 0.001). Comparisons of PRVR frequencies across demographic and modifiable lifestyle factors are presented in Table 2, along with data for symptom frequency and severity. In summary, females reported a higher frequency than males for both doses (*p* < 0.001). PRVR frequency was observed to decrease with increased age and body mass for both doses (*p* < 0.05). Those identifying as Asian reported the highest frequency of PRVR followed by those identifying as Black, Hispanic, and White in descending order (*p* < 0.05). There were no significant differences in PRVR among different blood types. 

Regarding modifiable lifestyle factors (Table 2), those reporting as non/never-smokers and non-alcohol users were observed to have a higher PRVR frequency compared to those reporting smoking or alcohol use (*p* < 0.05). Those performing planned physical exercise 2–3 days per week reported a higher incidence of PRVR following dose 1 compared to 0–1 days or 4+ days per week (*p* < 0.05), while those exercising >1 day per week reported an overall higher incidence of PRVR following dose 2 (*p* < 0.05). This observation was similar regarding the frequency of both resistance exercise and aerobic exercise. Lastly, 19.2% and 22.5% of the total patient population reported preventatively taking OTC pain medications immediately before or after the first and second vaccine doses, respectively, and reported a higher incidence of PRVR compared to those who did not report taking any preventative medications (*p* < 0.001). 

Regarding symptom frequency and severity (Table 2), the overall incidence of all PRVR symptoms was increased for dose 2, with the exception of “fatigue” (*p* < 0.05). Among those reporting PRVR, dose 2 yielded higher ratings of symptom severity for injection site pain and fatigue, while dose 1 yielded higher ratings for body ache, fever, headache, and joint pain (*p* < 0.05). In addition, age (r = −0.305), preventative OTC pain medications (r = 0.125), and dose number (r = 0.107) were significantly correlated with PRVR severity (averaged across all symptoms) (*p* < 0.001). Within the study population, 11.5% of participants (*n*= 1476) reported having previously contracted COVID-19. Those who had previously contracted COVID-19 reported significantly higher frequencies of PRVR following both dose 1 (48.5% vs. 32.1%, *p* < 0.05) and dose 2 (55.1% vs. 41.2%, *p* < 0.05), with increased scores of symptom severity following both doses among those reporting PRVR (*p* < 0.05). 

## 4. Discussion

The purpose of this investigation was to determine the risk factors associated with adverse effects after COVID-19 vaccination. While the World Health Organization (WHO) no longer considers COVID-19 a global health emergency, over one third of the global population is not fully vaccinated [2]. Determining the factors associated with vaccine reactivity can help to inform public health strategies to counteract vaccine hesitancy. Furthermore, understanding vaccine hesitancy and who is more at risk for developing adverse effects is not only relevant to COVID-19, but can also serve as a framework for public health initiatives and preparedness for future pandemics and vaccines. 

Our results suggest that COVID-19 vaccine reactivity is associated with several demographic and lifestyle factors. As the vaccine works through an mRNA-induced humoral immune response, patients with a more robust immune system may have more pronounced effects from the vaccine. Lifestyle factors such as drinking, smoking, and obesity have been shown to negatively impact the immune system, whereas physical activity and exercise have been shown to upregulate immune function [7,8,9,10]. Therefore, patients who are older, less active, and who consume alcohol or tobacco products may have a suppressed immune response to the vaccine, and may in turn experience less vaccine reactivity. While the degree to which PRVR may be indicative of a healthy immune response to vaccination is not fully established, our findings are consistent with, and add another layer to, the recent literature suggesting an association between symptoms after COVID-19 vaccination and anti-SARS-CoV-2 antibody response [11].

Interestingly, we found that the use of preventative OTC medications was paradoxically associated with an increased frequency of PRVR. It is unclear whether this association is mediated by an immunologic response to OTC medications, or by a psychological response, i.e., higher expectations and increased awareness of symptoms. The latter may be more likely as it is substantiated by the literature indicating a high rate of nocebo responses in placebo arms of COVID-19 vaccine trials [12].

Our results are consistent with the limited existing literature on factors associated with PRVR after the COVID-19 vaccine. One noteworthy study of 19,586 adults found that full vaccination dose, younger age, female sex, Asian ethnicity, and having had COVID-19 before vaccination were independently associated with an increased risk of adverse reactions [13]; variables such as body mass, physical activity level, and the use of preventative OTC medications were not evaluated. Of note, Asian ethnicity, as well as Black and Hispanic ethnicity, has been linked to increased COVID-19 vaccine hesitancy compared to White ethnicity [14]. While Asian ethnicity has not been linked to increased side effects after other vaccines, it has been linked to increased adverse effects after commonly prescribed medications due to pharmacogenetic differences in Asian populations [15]. Finally, we found that prior infection with COVID-19 was associated with an increased rate of PRVR frequency and severity, which has been described in the literature [16,17]. This may also be due to an increased immune response from prior COVID-19 infection leading to a more robust immune response to the vaccine [16].

This study is not without limitations. It was conducted through a single hospital system in the Texas Medical Center, which may limit its generalizability to all populations both within and outside of the United States. However, the study population was diverse in terms of age, sex, and ethnicity (Table 1). As the study was conducted electronically, not all participants responded to the survey. This could contribute to selection bias as participants who had adverse effects could be more likely to respond. While this limits the accuracy of the incidence of PRVR reported in this study, it likely has less of an effect on the association between the variables studied and PRVR. Finally, for those reporting to have previously contracted COVID-19, we were unable to collect information on the duration between when they were infected and when they received the vaccine. Further research is needed to more clearly determine the relationship between prior COVID-19 infection and PRVR. 

## 5. Conclusions

PRVR responses to the COVID-19 vaccine may be affected by several modifiable and non-modifiable factors that influence immune function. Further research is needed to determine whether these factors influence vaccine efficacy. 

## Figures and Tables

**Table 1:** Demographics, comorbidities, and modifiable lifestyle factors.

Demographics	Mean ± SD *
Sex	M = 5672, F = 7132, Not Specified = 86
Age (years)	63.5 ± 14.5 (18–98)
Height (cm)	169.5 ± 11.7
Weight (kg)	82.7 ± 40.9
BMI (kg/m^2^)	28.8 ± 6.8
Race	Frequency
White	77.5% (9993)
Black	6.6% (846)
Hispanic	7.6% (973)
Asian	4.8% (614)
Multiple Ethnicities	1.3% (164)
Other	2.3% (300)
Comorbidities	Frequency
Type 1 Diabetes	1.3% (171)
Type 2 Diabetes	12.7% (1631)
Diagnosed Cardiopulmonary Conditions	9.5% (1223)
Previous Heart Attack	4.1% (522)
Previous Stroke	2.9% (370)
Hypertension	35.9% (4631)
Hypothyroidism	13.1% (1693)
Hyperlipidemia/High Cholesterol	16.5% (2131)
Rheumatoid Arthritis	4.0% (521)
Asthma	8.1% (1038)
Cancer	7.1% (919)
Other Autoimmune Conditions	6.9% (887)
Modifiable Lifestyle Factors	Frequency
*Smoking*	
Never	67.4% (8692)
Former	28.4% (3658)
Current	3.7% (477)
Not Specified	0.5% (63)
*Alcohol Consumption*	
0 Drinks Per Day	65.2% (8409)
1-2 Drinks Per Day	30.1% (3873)
3-4 Drinks Per Day	3.6% (463)
5+ Drinks Per Day	0.5% (65)
Not Specified	0.6% (80)
*Physically Active/Exercise*	
Yes	69.1% (8906)
No	30.3% (3911)
Not Specified	0.6% (73)

* Survey response data are provided as means ± standard deviation (SD) for study population demographics as well as frequencies for race demographics, comorbidities, and modifiable lifestyle factors.

**Table 2:** Factors associated with patient-reported vaccine reactivity.

**PRVR Frequency—Comparison by Population Demographics**										
Comparisons of Interest	Dose 1					Dose 2				
Sex (male vs female)	Male	Female				Male	Female			
	23.7% (OR: 1.00)	41.8% * (OR: 1.76)				32.2% (OR: 1.0)	50.2% * (OR: 1.56)			
Age (years)	18–34	35–54	55–74	75+		18–34	35–54	55–74	75+	
	52.6% a (OR: 1.00)	51.3% a (OR: 0.97)	31.2% b (OR: 0.59)	17.5% c (OR: 0.32)		65.4% a (OR: 1.00)	61.0% a (OR: 0.93)	41.1% b (OR: 0.62)	24.7% c (OR: 0.37)	
Body Mass (kg)	<60 kg	60–79 kg	80–99 kg	100–119 kg	>120 kg	<60 kg	60–79 kg	80–99 kg	100–119 kg	>120 kg
	41.6% a (OR: 1.00)	36.4% ab (OR: 0.87)	32.0% b (OR: 0.77)	31.0% b (OR: 0.75)	29.9% b (OR: 0.72)	48.6% a (OR: 1.00)	44.2% b (OR: 0.91)	39.5% c (OR: 0.81)	39.5% c (OR: 0.81)	39.2% ^c^ (OR: 0.81)
Ethnicity	Asian	Black	Hispanic	White		Asian	Black	Hispanic	White	
	43.0% a (OR: 1.00)	42.6% ab (OR: 0.98)	37.3% b (OR: 0.82)	32.5% c (OR: 0.67)		52.9% a (OR: 1.00)	49.5% b (OR: 0.92)	43.1% c (OR: 0.76)	40.6% d (OR: 0.70)	
**PRVR Frequency—Comparison by Modifiable Lifestyle Factors**										
Comparsions of Interest	Dose 1					Dose 2				
Smoking	Current	Former	Never			Current	Former	Never		
	32.7% ab (OR: 0.88)	27.2% a (OR: 0.74)	37.0% b (OR: 1.00)			37.0% a (OR: 0.82)	35.9% a (OR: 0.80)	45.1% b (OR: 1.00)		
Alcohol (drinks/day)	None	1–2	3+			None	1–2	3+		
	36.2% a (OR: 1.00)	31.4% b (OR: 0.87)	21.3% c (OR: 0.59)			43.3% a (OR: 1.00)	40.8% b (OR: 0.94)	34.7% c (OR: 0.80)		
Planned Physical Exercise (days/week)	0–1	2–3	4+			0–1	2–3	4+		
	30.0% a (OR: 1.00)	38.0% b (OR: 1.07)	33.0% a (OR: 1.00)			39.0% a (OR: 1.00)	47.0% b (OR: 1.07)	44.0% b (OR: 1.08)		
Resistance Exercise (days/week)	0–1	2–3	4+			0–1	2–3	4+		
	34.2% a (OR: 1.00)	36.5% a (OR: 1.07)	35.3% a (OR: 1.03)			41.6% a (OR: 1.00)	46.0% b (OR: 1.07)	45.3% b (OR: 1.08)		
Aerobic Exercise (days/week)	0–1	2–3	4+			0–1	2–3	4+		
	33.2% a (OR: 1.00)	37.5% b (OR: 1.13)	33.0% a (OR: 0.99)			40.0% a (OR: 1.00)	46.3% b (OR: 1.16)	44.4% b (OR: 1.10)		
Preventative OTC Meds Immediately Before or After Dose (yes/no)	No	Yes				No	Yes			
	31.5% (OR: 1.00)	44.8% * (OR: 1.42)				39.6% (OR: 1.00)	51.1% * (OR: 1.29)			
**Symptom Frequency and Severity (VAS 0–100)**										
Frequency %Total Population	Inj. Pain	Body Ache	Nausea	Fever	Fatigue	Headache	Joint Pain			
Dose 1	28.8%	18.5%	11.3%	12.3%	22.4%	17.4%	13.4%			
Dose 2	33.4% *	27.4% *	15.8% *	19.3% *	24.4%	32.0% *	19.0% *			
Symptom Severity (VAS, 0–100) Among those reporting 1 or more symptoms	Inj. Pain	Body Ache	Nausea	Fever	Fatigue	Headache	Joint Pain	Average Symptom Severity		
Dose 1	36.2 ± 0.8	30.0 ± 0.9	6.4 ± 0.4	15.3 ± 0.7	24.0 ± 0.9	41.9 ± 0.9	15.1 ± 0.7	24.1 ± 0.8		
Dose 2	41.5 ± 1.2 *	20.6 ± 1.2 *	4.6 ± 0.6	8.6 ± 0.8 *	32.2 ± 1.5 *	18.6 ± 1.2 *	10.8 ± 1.0 *	19.5 ± 1.1 *		
**PRVR Frequency and Severity—Previously Contracted COVID-19 Prior to Vaccination Dose 1**										
Frequency %Total Population	Prior COVID-19			No Previous Infection						
Dose 1	48.54% * (OR: 1.51)			32.11% (OR: 1.00)						
Dose 2	55.14% * (OR: 1.34)			41.22% (OR: 1.00)						
Symptom Severity (VAS, 0–100) Among those reporting 1 or more symptoms	Inj. Pain	Body Ache	Nausea	Fever	Fatigue	Headache	Joint Pain	Average Symptom Severity		
Dose 1										
Prior COVID-19	43.4 ± 3.2	27.9 ± 3.1	6.3 ± 1.7	17.1 ± 2.9	40.5 ± 3.7	25.0 ± 3.3	14.1 ± 2.8	24.9 ± 2.0		
No Previous Infection	41.2 ± 1.4	19.0 ± 1.3 *	4.3 ± 0.6 *	6.8 ± 0.8 *	30.3 ± 1.6 *	17.1 ± 1.3 *	10.1 ± 1.1 *	18.4 ± 0.7 *		
Dose 2										
Prior COVID-19	38.2 ± 2.2	35.4 ± 2.5	8.5 ± 1.4	20.3 ± 2.2	30.1 ± 2.5	46.9 ± 2.6	19.7 ± 2.2	28.4 ± 1.5		
No Previous Infection	35.9 ± 0.8	29.1 ± 0.9 *	6.1 ± 0.5 *	14.4 ± 0.7 *	23.0 ± 0.9 *	41.1 ± 1.0 *	14.4 ± 0.8 *	23.4 ± 0.5 *		

Survey response data for patient-reported vaccine reactivity (PRVR) are provided as frequencies for subgroup breakdowns by demographics, modifiable lifestyle factors, and specific symptom frequency. Data are also provided as means ± 95%CI for symptom severity ratings reported using a visual analog scale (VAS: 0–100). For single pairwise comparisons, * = significant difference between groups at *p* < 0.05. For variables involving multiple comparisons, differing letters between groups are significantly different at *p* < 0.05. For PRVR data, odds ratios (OR) are also provided. Inj. Pain = injection site pain.

## Data Availability

The data presented in this study are available on request from the corresponding author.
